# Intraspecific competition and light effect on reproduction of *Ligularia virgaurea*, an invasive native alpine grassland clonal herb

**DOI:** 10.1002/ece3.975

**Published:** 2014-02-20

**Authors:** Tian-peng Xie, Ge-fei Zhang, Zhi-gang Zhao, Guo-zhen Du, Gui-yong He

**Affiliations:** State Key Laboratory of Glassland and Agro-Ecosystems, School of Life Sciences, Lanzhou UniversityLanzhou, 730000, China

**Keywords:** Clonal growth, intraspecific competition, light, *Ligularia virgaurea*, sexual reproduction, threshold size

## Abstract

The relationship between sexual reproduction and clonal growth in clonal plants often shows up at the ramet level. However, only a few studies focus on the relationship at the genet level, which could finally account for evolution. The sexual reproduction and clonal growth of *Ligularia virgaurea*, a perennial herb widely distributed in the alpine grasslands of the Qinghai-Tibetan Plateau of China, were studied under different competition intensities and light conditions at the genet level through a potted experiment. The results showed that: (1) sexual reproduction did not depend on density or light, and increasing clonal growth with decreasing density and increasing light intensity indicated that intraspecific competition and light intensity may affect the clonal life history of *L. virgaurea*; (2) both sexual reproduction and clonal growth show a positive linear relationship with genet size under different densities and light conditions; (3) a threshold size is required for sexual reproduction and no evidence of a threshold size for clonal growth under different densities and light conditions; (4) light level affected the allocation of total biomass to clonal and sexual structures, with less allocation to clonal structures and more allocation to sexual structures in full sunlight than in shade; (5) light determined the onset of sexual reproduction, and the genets in the shade required a smaller threshold size for sexual reproduction to occur than the plants in full sunlight; and (6) no evidence was found of trade-offs between clonal growth and sexual reproduction under different densities and light conditions at the genet level, and the positive correlation between two reproductive modes indicated that these are two integrated processes. Clonal growth in this species may be viewed as a growth strategy that tends to maximize genet fitness.

## Introduction

Many perennial plants multiply through both sexual reproduction and clonal growth; ecologists focused on the relationship between the two reproduction processes for many years (Schmid et al. 1995; Van Kleunen et al. 2001; Eckert 2002; Brown and Eckert 2005). The two reproduction processes have obvious differences in terms of their diffusion distance, offspring production, and establishment success rate (Winkler and Fischer 2001). At present, many ecologists evaluated the resource trade-offs between sexual and clonal reproductive allocation in clonal plants (Hartemink et al. 2004; Thompson and Eckert 2004; Bai et al. 2009; Liu et al. 2009), the majority of these studies have focused on measuring trade-offs at the ramet level (ramet-derived), and only a few studies have investigated the trade-offs at the genet level (seed-derived) (reviewed in Vallejo-Marin et al. 2010). Interestingly, no direct resource-based trade-offs between sexual and clonal reproduction have been found at the genet level (Cain and Damman 1997; Mendoza and Franco 1998; Thiele et al. 2009). This finding may be either because the two reproduction processes may be affected by different resources (thus, adding reproductive input in one process does not necessarily lead to a reduction in the another) (Cain and Damman 1997; Reekie 1999) or because of the physiological integration between genet and ramets, clonal plant could use resources efficiently and, ultimately, clonal growth promotes sexual reproduction (Mendoza and Franco 1998). However, a basic condition must be satisfied in terms of division of labor; the resources can be transferred in both directions between genet and ramets. One of the examples of trade-offs between clonal and sexual reproduction at the genet level is in *Sagittaria latifolia* (Van Drunen and Dorken 2012) because short-lived spacers limit the division of labor and resource sharing between ramets.

The different resource allocation of clonal plants may be an adaptive strategy of plasticity to different environments. The variation in resource allocation to clonal growth versus sexual reproduction may be environmental condition (Liu et al. 2009), population density (Nishitami et al. 1999;Ronsheim and Bever 2000; Van Kleunen et al. 2001), plant size (Hartnett 1990; Schmid et al. 1995), age (Lopez et al. 2001), and genetics (Bostock 1980; Reekie 1991; Prati and Schmid 2000; Ronsheim and Bever 2000). In a clonal plant population, plant density plays an important role in life-history evolution. Population density increases due to clonal growth, which leads to higher intraspecific competition and, in turn, can change the allocation of resources to clonal growth and sexual reproduction; it thus affects fitness, genet size, reproductive values, population size, and genetic structure (Heywoods 1986). Intraspecific competition may refer to both competition among genets and ramets; however, what finally accounts for evolution is competition at the genet level (Van Kleunen et al. 2001). According to a lattice structure model, Ikegami et al. (2012) found that the production of ramets at lower densities and the production of seeds at higher densities seem to be a proper strategy because, when sexual reproduction is given higher relative allocation under high density, seed dispersal may act as an escape mechanism from the unfavorable site (Abrahamson 1980; Gardner and Mangel 1999; Van Kleunen et al. 2001; Rautiainen et al. 2004). However, increasing resource allocation to clonal growth may make the genet more competitive in high-density populations (Williams et al. 1977; Loehle 1987). Nevertheless, all the studies were conducted at the ramet level; whether trade-offs exist between the two reproduction processes under different densities at the genet level is unknown.

Light is one of the most important environmental factors that influence the allocation of growth and biomass in plant species (Wang et al. 2008). Variations in light intensity could create strong selection pressures and affect leaf morphology and biomass allocation among roots, stems, and leaves (Curt et al. 2005; Wang et al. 2008). However, the effects of light intensity on the relationship between clonal and sexual structures are unknown. Abrahamson (1980) reported that clonal plants tend to allocate proportionally more biomass to clonal growth and less to sexual reproduction under growth-limiting conditions. However, Gardner and Mangel (1999) predicted that favorable habitats should promote clonal growth over sexual reproduction. Different reproductive modes might suggest different adaptive strategies of plants to varying environments. So the effects of the light levels on the relationship between two reproductive processes are worth studying.

Given the plasticity of plant growth (Harper 1977), individuals have different sizes in natural population. Weiner (1988) predicted a linear relationship between plant size and sexual reproduction output and that plants should have a minimum size for reproduction. The pattern was observed in many cases (Hartnett 1990; Schmid and Weiner 1993; Schmid et al. 1995; Van Zandt et al. 2003). Sanson and Werk (1986), and Rees and Crawley (1989) also found a linear relationship between plant size and reproduction, but they did not find a threshold size for reproduction. Zhao et al. (2004) also found no evidence of a threshold size requirement for reproduction in five buttercup species. In many cases, no threshold size exists between genet size and clonal growth (Eriksson 1985; Hartnett 1990; Méndez and Obeso 1993; Schmid et al. 1995). However, Pluess and Stöcklin (2005) found that a threshold size exists for the clonal reproduction of *Geum reptans*. Recently, Weiner et al. (2009) summarized the relationship between individual vegetative (V, *x*-axis) and reproductive biomass (R, *y*-axis) in 76 species, and they found that the clonal plants of those species usually show a simple, linear relationship, which either (1) pass through the origin or (2) with a positive *x*-intercept.

In this study, we investigated the effects of intraspecific competition and light intensity on two *Ligularia virgaurea* reproduction processes at the genet level through a potted experiment. We test the following hypotheses: (1) More resource should be allocated to sexual reproduction in high-density environments or in shade, whereas clonal growth should dominate in low-density environments or in full natural irradiance; (2) both sexual reproduction and clonal growth show a positive linear relationship with genet size under different densities and light conditions; (3) both sexual reproduction and clonal growth require an initial investment, measurable as a positive minimum size for the two processes under different densities and light conditions; and (4) no trade-offs exist between sexual reproductive allocation and clonal reproductive allocation under different densities and light conditions at the genet level.

## Materials and Methods

### Study species

*Ligularia virgaurea* (Maxim.) is a typical, naturally occurring, native, noxious weed that belongs under *Ligularia* Cass. in Compositae. *L. virgaurea* is widely distributed in the alpine grasslands of the Tibetan Plateau in China and is an herbaceous perennial plant characterized by clonal reproduction through rhizomes and sexual reproduction. The life cycle of *L. virgaurea* is very long. Moreover, it has no stems and is comprised only of several leaves at the vegetative stage. *L. virgaurea* produces a flowering stem at the sexual production stage after 3–6 years. The plasticity of its rhizome is very strong; thus, it is a typical guerilla-type plant (Shan et al. 2000). *L. virgaurea* can extend successfully and become an important component of the alpine plant communities in many habitats, especially in meadows heavily disturbed by grazing. Some noxious succi are observed in its roots, stem, and leaves, which emanate a scent that deters livestock from eating them. Thus, it is one of the most serious weeds that infest the alpine meadows on the Tibetan Plateau.

### Experiment and measurements

The potted experiment was conducted at the Alpine Meadow Ecosystem Scientific Research Station of Lanzhou University based in Hezuo (34°55′N, 102°53′E, Hezuo County, Gannan, Gansu Province, China), with an average altitude of over 2900 m a.s.l. The mean daily air temperature is 2.0°C, which ranges from −8.9°C in January to 11.5°C in July. Mean annual precipitation is 550 mm (Du and Wang 1995).

Two light treatments are available: full sun (1378.57 ± 79.69 *μ*mol m^−2^ s^−1^) and 75% natural irradiance (893.71 ± 25.12 *μ*mol m^−2^ s^−1^) (which correspond to the mean intensity of light captured by this species in the natural community) that were realized by covering with a canopied black nylon net above the pots and two planting density treatments (high density and low density) in the potted experiment. Seeds used for the experiment were collected randomly from more than 15 populations in September and October 2007 at Maqu station (33°45′N, 102°04′E, Maqu County, Gannan, Gansu Province, China, average altitude is over 3500 m a.s.l.). The seeds were cleaned, mixed well, and stored in paper bags in the dark at laboratory temperature. In early May 2008, the plastic pots (35 cm diameter, 25 cm deep) were filled with loam soil obtained from Hezuo station (the nutrient element contents of soil C: 0.68 ± 0.08 g/kg; N:0.27 ± 0.04 g/kg; P:0.66 ± 0.14 g/kg; PH: 6.27 ± 0.27). Each pot included 60 seeds that were buried into a depth of 1 cm. Given that *L. virgaurea* begins clonal growth in the second growing season (Wang et al. 2008), each pot had ≤5 plants (low density, ≈15/m^2^) and ≥35 plants (high density, ≈100/m^2^) at each light treatment at the beginning of the second growing season (in mid-May 2009). The densities treatment depended on the investigation of the natural population (low density, 18.33 ± 8.17/m^2^; high density, 75.44 ± 26.48/m^2^). Each treatment contained 40 pots, and they were arranged randomly at the same light condition. Pots were hand-weeded and hand-watered every year, and no fertilizer was applied throughout four growing seasons (from 2008 to 2011). Given that *L. virgaurea* begins sexual reproduction in the fourth growing season in this experiment, we sampled in mid-July 2011. We selected 10 pots with high density in each light condition (100% *N* = 405; 75% *N* = 416) because of the larger samples, and 40 pots with low density in each light condition (100% *N* = 196; 75% *N* = 171). We used scissors to cut the edges of pots and washed away the soil with a high-pressure jet of water. Every genet (including its ramets, rhizomes, and other structures, which we washed carefully to avoid breakage of rhizomes) was then cleaned and placed in a large envelope. Distinguishing genet and ramets is easy considering their different sizes. We measured the proportion of genets with clonal growth and sexual reproduction, number of rhizomes per genet, number of flower heads per genet, genet biomass (roots + leaves + stem), mass of clonal structures (rhizomes + ramets), mass of sexual structures (flower heads), RE of clonal growth, and sexual reproduction.

### Statistical analyses

All analyses were performed using SPSS for Windows version 16.0 (SPSS Inc., Chicago, IL). We used two-way ANOVA to determine the effects of density and light on genet biomass, proportion of clonal reproduction, number of rhizomes, mass of clonal structures, RE of clonal structures, proportion of sexual reproduction, number of flower heads, mass of sexual structures, and RE of sexual structures in the pot experiment; Tukey's post hoc analysis method was used.

We investigated the relationship between genet size, sexual reproduction and clonal growth using the simple model (Weiner 1988; Schmid et al. 1995) for the mass of sexual structures *r* = *b*_r_*(*v*-*a*_r_) and the mass of clonal structures *c* = *b*_c_*(*v*-*a*_c_), where *v* is genet biomass, *a*_r_ and *a*_c_ are the minimum size required for sexual reproduction and clonal growth, respectively, and *b*_r_ and *b*_c_ are the slope parameters for the two relationships. We used analysis of covariance (ANCOVA) to examine the variation in the mass of clonal structures, sexual structures and genet biomass with density or light as fixed effects, and the genet biomass as a covariate.

We used the partial correlation analysis to investigate the relationship between the mass of clonal structures and the mass of sexual structures controlled by genet mass.

## Results

The genet biomass (*F*_1,1186_ = 27.453, *P* < 0.0001), proportion of clonal reproduction (*F*_1,1186_ = 27.816, *P* < 0.0001), and number of rhizomes (*F*_1,1186_=31.215, *P* < 0.0001) were significantly greater in low density than in high density, Nevertheless, density did not significantly affect the mass of clonal structures (*F*_1,475_=2.226, *P* = 0.157), RE of clonal structures (*F*_1,475_=3.562, *P* = 0.063), the proportion of sexual reproduction individuals (*F*_1,1186_=1.174, *P* = 0.279), number of flower heads (*F*_1,46_=2.110, *P* = 0.153), mass of sexual structures (*F*_1,46_=0.027, *P* = 0.871), and RE of sexual structures (*F*_1,46_=2.674, *P* = 0.109) (Table 1). These indicate that the clonal reproduction of *L. virgaurea* depended on density, whereas sexual reproduction does not.

**Table 1:** Variations in proportion of clonal and sexual reproduction individuals, number of rhizomes, number of flower heads, genet biomass, mass of clonal and sexual structures, and RE of clonal and sexual structures in *Ligularia virgaurea* on two factors (density and light treatments)

	Full sun		75% of natural irradiance	
	High density	Low density	High density	Low density
Genet biomass (g)	2.76 ± 0.15^a^	4.44 ± 0.33^b^	2.25 ± 0.13^c^	2.71 ± 0.21^a^
Proportion of clonal reproduction (%)	46.06 ± 2.51^a^	63.30 ± 3.50^b^	32.60 ± 2.30^c^	48.12 ± 4.02^a^
Number of rhizomes	1.01 ± 0.07^a^	1.56 ± 0.14^b^	0.58 ± 0.05^c^	1.03 ± 0.11^a^
Mass of clonal structures (g)	0.28 ± 0.01^a^	0.30 ± 0.02^a^	0.20 ± 0.01^b^	0.22 ± 0.01^b^
RE of clonal structures (%)	10.89 ± 0.86^a^	9.47 ± 0.78^ab^	8.84 ± 0.61^ab^	6.83 ± 1.05^b^
Proportion of sexual reproduction (%)	4.15 ± 0.99^a^	3.57 ± 1.33^a^	4.69 ± 1.03^a^	2.55 ± 1.26^a^
Number of flower heads	19.60 ± 2.40^a^	15.43 ± 2.17^a^	19.59 ± 1.44^a^	16.88 ± 3.07^a^
Mass of sexual structures (g)	1.05 ± 0.12^a^	1.27 ± 0.15^a^	0.62 ± 0.12^b^	0.73 ± 0.28^b^
RE of sexual structures (%)	11.61 ± 1.16^a^	8.73 ± 1.58^a^	9.03 ± 1.01^a^	6.74 ± 1.15^a^

The values in the table are mean ± SE. Means of traits that do not share superscripts are significantly different at the *P* < 0.05 level according to Tukey's test.

The genet biomass (*F*_1,1186_=22.688, *P* < 0.0001), proportion of clonal reproduction (*F*_1,1186_=26.885, *P* < 0.0001), number of rhizomes (*F*_1,1186_=34.966, *P* < 0.0001), mass of clonal structures (*F*_1,475_=26.314, *P* < 0.0001), RE of clonal structures (*F*_1,475_=7.237, *P* = 0.007), and mass of sexual structures (*F*_1,46_=7.474, *P* = 0.009) were significantly greater in full sunlight than in shade. However, light intensity did not significantly affect the proportion of sexual reproduction individuals (*F*_1,1186_=0.019, *P* = 0.891), number of flower heads (*F*_1,46_=1.069, *P* = 0.307), and RE of sexual structures (*F*_1,46_=2.661, *P* = 0.110) (Table 1). These indicate that light intensity facilitates the genet growth and the clonal reproduction of *L. virgaurea,* whereas the sexual reproduction does not.

**Figure 1:**
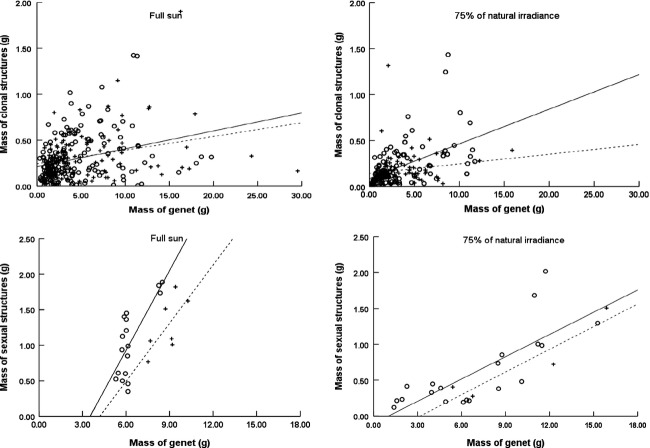
Relationship between the mass of clonal or sexual structures and mass of genet at harvest for Ligularia virgaurea on two factors (density and light treatments) (high: ○,-; low: +,…).

Clonal reproduction and sexual reproduction were positively related to genet mass in two factors (density and light level). However, the regression lines for the size dependence of sexual reproduction and clonal growth differ in their *x*-intercepts. The data suggest that a positive minimum genet size had to be attained for sexual reproduction to begin, but no evidence exists for a minimum size for clonal growth (Fig. 1, Table 2). We found that the genets with sexual reproduction must be accompanied by clonal growth, whereas genets with clonal growth were not necessarily accompanied by sexual reproduction.

**Table 2:** Parameter estimates for the relationship between the mass of clonal structures or sexual structures and genet biomass at harvest for *Ligularia virgaurea* on two factors (density and light treatments) (^*^P < 0.05, ^*^^*^^*^P < 0.0001)

Treatments	*y*-intercept (±SE)	Slope (±SE)	*n*	*R*^2^
Clonal structures				
Full sun				
High density	0.210 ± 0.028	0.020 ± 0.005^*^^*^^*^	162	0.085
Low density	0.242 ± 0.036	0.015 ± 0.005^*^^*^^*^	107	0.083
75% of natural irradiance				
High density	0.085 ± 0.022	0.038 ± 0.005^*^^*^^*^	123	0.289
Low density	0.168 ± 0.015	0.022 ± 0.003^*^^*^^*^	85	0.025
Sexual structures				
Full sun				
High density	−1.320 ± 0.555	0.374 ± 0.087^*^	17	0.554
Low density	−1.143 ± 1.136	0.272 ± 0.128^*^	7	0.476
75% of natural irradiance				
High density	−0.104 ± 0.162	0.103 ± 0.020^*^^*^^*^	20	0.592
Low density	−0.334 ± 0.321	0.105 ± 0.029^*^	4	0.865

The allocation rate of biomass to genets and clonal structures did not significantly differ between high-and low-density plants in both light conditions (*F* = 0.463, *P* = 0.497 in full sunlight and *F* = 3.204, *P* = 0.064 in 75% natural irradiance as a comparison of regression slopes). The allocation rate of biomass to sexual reproduction in both light conditions also did not significantly differ between high-and low-density plants in both light conditions (*F* = 0.383, *P* = 0.543 in full sunlight and *F* = 0.002, *P* = 0.968 in 75% natural irradiance).

The plants in full sunlight allocate less biomass to clonal growth and more biomass to sexual reproduction than the plants under 75% natural irradiance with high density (Fig. 1, Table 2) (clonal growth: *F* = 5.485, *P* = 0.020; sexual reproduction: *F* = 9.201, *P* = 0.005). However, this tendency was not significant in low density (clonal growth: *F* = 0.180, *P* = 0.672; sexual reproduction: *F* = 1.750, *P* = 0.227). This finding indicates that the plants in full sunlight tended to allocate a comparatively smaller proportion of biomass to clonal structures and a higher proportion of biomass to sexual structures than the shaded plants.

The different threshold sizes for sexual reproduction to occur were not significant between the two densities in both light conditions (Fig. 1, Table 2) (*F* = 2.163, *P* = 0.156 in full sunlight and *F* = 0.340, *P* = 0.566 in 75% natural irradiance as a comparison of *y*-intercepts), but the plants in full sunlight required a larger threshold size for sexual reproduction to occur than the shaded plants in both densities (Fig. 1, Table 2) (*F* = 11.029, *P* = 0.002 in high density and *F* = 8.081, *P* = 0.022 in low density for comparison of *y*-intercepts). These indicate that light condition, rather than density, affected the onset of sexual reproduction for *L. virgaurea*.

According to the partial correlation analysis controlled by genet mass, a positive correlation tendency was found between mass of clonal structures and mass of sexual structures in high density, regardless of whether the plant was in full sunlight (*P* = 0.070, *R* = 0.464, df = 14) or in shade (*P* = 0.067, *R* = 0.429, df = 17). This tendency was significant in low density under full sunlight (*P* = 0.050, *R* = 0.812, df = 4), but not significant in 75% natural irradiance (*P* = 0.642, *R* = 0.533, df = 1). These indicate that no trade-offs exist between two reproductive processes.

## Discussion

### Density and Light Dependency of Reproduction in *Ligularia virgaurea*

The lattice structure model by Ikegami et al. (2012) showed that the production of ramets at lower densities and the production of seeds at higher densities seem to be a proper strategy. Some practical researches support the predictions of this model (Abrahamson 1980; Gardner and Mangel 1999; Van Kleunen et al. 2001; Rautiainen et al. 2004). However, the reproductive density response in *L. virgaurea* is the consequence of constant sexual reproductive allocation and increasing clonal reproductive allocation with decreasing density. We found that only some earlier studies show evidence that sexual reproductive allocation does not depend on density (Ogden 1974; Thomas 1974; Holler and Abrahamson 1977). According to the model by Williams (1975), to achieve genetic survival, the clone must disperse genetically variable propagules beyond its current area. Although the cost of sexual reproduction is much higher than that of asexual reproduction, especially while the clone is spreading, a balance is achieved whereby sexuality becomes evolutionarily valuable to avoid the wasted expenditure of producing genetically identical individuals in an area of increasing density and thus intensified competition (Holler and Abrahamson 1977). Our results follow the models by Ikegami et al. (2012), which exhibit increased clonal growth when population density is low and clonal expansion is possible, which is a low-risk strategy that facilitates rapid local spread.

Despite differences in the genet biomass, light intensity could influence the clonal growth strategies of *L. virgaurea,* but it has little effect on sexual reproductive strategies (it only has a significant effect on the absolute investment of sexual reproduction). The shaded condition restrained the accumulation of genet biomass and clonal growth and increased the cost of clonal growth. Thus, it affected the continuation of the entire clonal population. In our experiments, the shade treatment simulated the light intensity in the natural population of *L. virgaurea,* and the full sunlight treatment simulated the overgrazing environment where the *L. virgaurea* have sufficient light resources and their clonal structures dominate in the process of capturing and using light. Therefore, the change in biomass allocation to clonal structures in light heterogeneous habitats is an evolutionary adaptation strategy of *L. virgaurea*.

### Size Dependency of Reproduction in *Ligularia virgaurea*

The linear size dependency model worked well for both sexual reproduction and clonal growth. We found that a minimum genet size should be attained for sexual reproduction, but no evidence for such requirement exists for clonal growth in different densities (Fig 1). In the potted experiment, the onset of clonal reproduction in *L. virgaurea* takes place at the second growing season (Wang et al. 2008), but the onset of sexual reproduction takes place at the fourth growing season. Meanwhile, the genets with sexual reproduction must be accompanied by clonal ramets, but the genets with clonal growth did not necessarily have a chance to sexually reproduce. Schmid et al. (1995) considered that rhizome growth in a clonal perennial species is parallel to branch growth in a highly branched species, and no threshold size may exist for these activities if they are part of a single growth process. Moreover, sexual reproductive structures were the last plastic structures produced by plants. A plant cannot commit itself to producing these structures until it has accumulated a certain amount of resources. Thus, sexual reproduction clearly requires the achievement of a threshold size, whereas clonal growth does not.

We found that light level, rather than density, is the factor that influences the allocation of total biomass to clonal or sexual structures. The genets allocated less biomass to clonal structures and more biomass to sexual reproduction under full natural irradiance than under shade. Thus, plants clonally and sexually reproduced with different strategies under different light conditions. Clonal growth was thought to enable plants to forage for light-rich microsites to facilitate the establishment of genets and decrease mortality risk under shade (Chu et al. 2006; Wang et al. 2008). Moreover, sexual reproduction was thought to enable plants to produce high-quality offspring under full sunlight.

Light levels, rather than density, primarily determine the onset of sexual reproduction. The plants in full sunlight required a larger threshold size for sexual reproduction to occur than the plants in shade. Thus, plants have a lesser genet size requirement for the onset of sexual reproduction under shade because plants have difficulty accumulating resources. For this species, the genet could adjust genet size for the onset of sexual reproduction according to different environments.

### Relationship between Sexual and Clonal Reproduction of *Ligularia virgaurea*

We found a positive correlation between sexual reproductive structures and clonal reproductive structures in high density under two light conditions. This tendency was significant in low density under full sunlight, but not significant under shade. The latter may be attributed to the smaller sample size (*N* = 4). We failed to detect trade-offs between two reproductive modes in this species. Trade-offs apparent at the ramet level may not scale up to the genet level, because the ramets within a genet do not all perform the same function, and costs incurred at the ramet level may not additively affect genet fitness (Vallejo-Marin et al. 2010). Commonly, only a few ramets are reproductive at once, and the cost of reproduction at the genet level might be compensated through the reallocation of resources within the genet (Mendoza and Franco 1998; Thiele et al. 2009).

In this species, the lack of evidence of trade-offs between two reproduction processes suggests that these are two integrated processes. Clonal plants with persistent rhizomes often have the ability to share resources among ramets and exhibit “clonal integration.” Indeed, we found that the spacers between genet and ramets still connect in the fourth growth season. The rhizomes belong to long-lived spacers, which ensure that the resources can be transferred in both directions between genet and ramets. Rhizomes that persist over multiple growing seasons have also been found in other studies (Mendoza and Franco 1998). Accordingly, clonal growth increases the size of the genet, which in turn enhances its reproductive potential; clonal growth should be viewed as a growth strategy that tends to maximize genet fitness (Mendoza and Franco 1998), and sufficient light and less intraspecific competition could promote the interdependence of two reproductive modes at the genet level in this species.

In conclusion, intraspecific competition and light levels may affect the genet size and the clonal life history of *L. virgaurea*, and reproduction is shifted toward the asexual mode at lower densities and under full natural irradiance. Our study suggests that intraspecific competition and light level are the important factors in the evolution of the clonal life history. Compared with intraspecific competition, light level has more influence on the variation of reproductive allocation at the genet level. The total biomass allocated less to clonal structures and more to sexual reproduction under full natural irradiance than under shade. Such life-history strategies help this species maintain its population sizes and decrease mortality in natural communities. These strategies also help produce high-quality offspring to establish new settlements in meadows disturbed heavily by gazing. We conclude that the genets could not begin sexual reproduction unless they accumulate sufficient biomass, and the threshold size could adjust through light conditions. The lesser requirement of threshold size under shade enables them to produce new genotype offspring in competitive natural communities. No evidence was found of trade-offs between clonal growth and sexual reproduction at the genet level. The positive correlation between two reproductive modes indicated that these are two integrated processes, and the relationship can be promoted in meadows heavily disturbed by grazing with less intraspecific competition.
